# Cultural adaptation and validation of the Freiburg Life Quality Assessment - Wound Module to Brazilian Portuguese^1^

**DOI:** 10.1590/1518-8345.0289.2684

**Published:** 2016-05-03

**Authors:** Elaine Aparecida Rocha Domingues, Neusa Maria Costa Alexandre, José Vitor da Silva

**Affiliations:** 2 Doctoral student, Universidade Estadual de Campinas, Campinas, SP, Brazil. Assistant Professor, Escola de Enfermagem Wenceslau Braz, Itajubá, MG, Brazil; 3 PhD, Associate Professor, Universidade Estadual de Campinas, Campinas, SP, Brazil; 4 PhD, Full Professor, Escola de Enfermagem Wenceslau Braz, Itajubá, MG, Brazil

**Keywords:** Wounds and Injuries, Quality of Life, Validity of Tests

## Abstract

**Objectives:**

to adapt the Freiburg Life Quality Assessment - Wound Module to Brazilian Portuguese and to measure its psychometric properties: reliability and validity.

**Method:**

the cultural adaptation was undertaken following the stages of translation, synthesis of the translations, back translation, committee of specialists, pre-test and focus group. A total of 200 patients participated in the study. These were recruited in Primary Care Centers, Family Health Strategy Centers, in a philanthropic hospital and in a teaching hospital. Reliability was assessed through internal consistency and stability. Validity was ascertained through the correlation of the instrument's values with those of the domains of the Ferrans and Powers Quality of Life Index - Wound Version and with the quality of life score of the visual analog scale.

**Results:**

the instrument presented adequate internal consistency (Cronbach alpha =0.86) and high stability in the test and retest (0.93). The validity presented correlations of moderate and significant magnitude (-0.24 to -0.48, p<0.0001).

**Conclusion:**

the results indicated that the adapted version presented reliable and valid psychometric measurements for the population with chronic wounds in the Brazilian culture.

## Introduction

Chronic wounds present delay in the physiological repair of the healing, that is, they enter into a pathological inflammatory state. These are wounds which last six weeks or over and present high rates of reoccurrences^(^ 1 ^-^ 2 ^)^. At the time of writing, they are considered a worldwide epidemic, found in approximately 1% of the adult population and 3.6% of individuals aged over 65 years old^(^ 2 ^)^.

As a result of this slowness in the healing process, and the high rate of recurrence, the individual with wounds may present physical, social, psychological and economic changes, negatively influencing the undertaking of her activities of daily living. These changes in the patient's day-to-day life have negative repercussions for her quality of life^(^ 3 ^-^ 4 ^)^.

As a result, in order to provide quality care, it is necessary for the health team to analyze the patient holistically, taking into account the aspects of the wound and of her quality of life. Although quality of life is subjective, there are numerous questionnaires available for evaluating it. 

The literature presents the Ferrans and Powers Quality of Life Index - Wound Version (FPQLI-WV), which measures the quality of life of patients with chronic and acute wounds^(^ 5 ^)^, the Charing Cross Venous Ulcer Questionnaire (CCVUQ)^(^ 6 ^)^ and the Venous Leg Ulcer Quality of Life questionnaire (VLU-QoL)^(^ 7 ^)^, which investigate the quality of life of individuals with venous ulcers. 

It is highlighted that the FPQLI-WV does not address a dimension related to the treatment of the wound, an important factor which significantly influences these patients' quality of life, as, according to studies, quality care represents a positive factor in quality of life^(^ 4 ^,^ 8 ^)^.

The CCVUQ and VLU-QoL instruments were developed only to be used in individuals with venous ulcers, limiting the assessment of the quality of life of patients with other etiologies of wounds. 

This being the case, the decision was made to undertake the cultural adaptation of the instrument termed the Freiburg Life Quality Assessment - Wound Module (FLQA-W) to Brazilian Portuguese. This instrument was chosen as it is short and easy to apply, as well as possessing satisfactory psychometric properties^(^ 9 ^)^.

The abbreviated version is known as the FLQA-Wk and aims to measure the quality of life of people with chronic wounds over the previous week. It is made up of 24 items, distributed in six domains: physical symptoms, daily life, social life, psychological well-being, treatment and satisfaction. It has been validated in three separate studies with individuals with acute and chronic wounds^(^ 9 ^)^.

It is hoped that this instrument's use in Brazil may viabilize a tool for the work of health professionals, particularly nurses, both in research and in the design of interventions, consequently improving the quality of life of patients with chronic wounds. 

As a result, the present study aimed to adapt the abbreviated version of the Freiburg Life Quality Assessment (FLQA)-Wound Module to Brazilian Portuguese, and to measure the psychometric properties of the adapted instrument: reliability and validity. 

## Method

This is a study with a quantitative approach, of the methodological type, grounded in the theoretical reference of cultural adaptation and validation. It followed the stages recommended by international studies^(^ 10 ^-^ 12 ^)^.

### The Procedure of Cultural Adaptation

First of all, the FLQA-Wk instrument was translated into Brazilian Portuguese by two independent translators. One nurse with knowledge in the area of wounds and of the study's objectives, and one professor of modern languages, participated in this phase. Each participant undertook one translation, totaling two independent versions (VT1 and VT2).

The analysis of the divergences between the versions was undertaken by the two translators, in conjunction with one of the researchers. Following this analysis, a version was developed for the back translation (VT12).

The back translation was undertaken by two translators whose native language was English and who had a good command of the Portuguese language. Neither was informed of the study's concepts and objectives, and they undertook the translations independently (R1 and R2).

All the versions (VT1, VT2, VT12, R1 and R2) were sent to a committee of eight judges who undertook an analysis relating to the semantic, idiomatic, cultural and conceptual equivalencies. The specialists were selected due to their knowledge about wounds, quality of life, or the translation of instruments. 

The validity of the instrument's content was investigated through calculating the Content Validity Index (CVI). This test evaluates the level of agreement among the judges on specific aspects of the adapted questionnaire and of its items. The judges scored the items with values ranging from one to four. For the study, a level of agreement equal or superior to 0.8 was stipulated^(^ 13 ^)^.

Subsequently, a pretest was undertaken with 30 subjects^(^ 11 ^)^ with chronic wounds of differing etiologies, attended in a wound treatment center. All were interviewed and were requested to talk about the difficulties found in answering the questionnaire. As some interviewees showed doubts in interpreting some questions, the decision was made to hold a focus group, a technique used in similar studies, with the objective of adapting the instrument's content to the specific population^(^ 14 ^-^ 15 ^)^.

A meeting was held with five patients with differing educational levels (two illiterates, two who had not finished their basic education, and one who had completed higher education). The focus group began with a description of the study and the importance of each individual's participation. The items of the questionnaire were presented separately, to make it easier for the participants to absorb the information. The focus group was run with the participation of a moderator and two observers, following steps recommended in the literature: assembly, conduct of the group, and analysis of the data^(^ 16 ^)^.

With the authorization of those present, the entire process was recorded. The moderator was responsible for reading the questionnaire out loud and for the discussion, while the observers analyzed all the issues raised. Following the reading of each item, the participants were questioned regarding its interpretation. In accordance with what people said, associated with the nonverbal expressions indicating doubt, the item was discussed, with a vote being held regarding modifications. The Final Translated Version (FTV) was then elaborated. 

Data were collected in nine Primary Care Centers (UBS) and eight Family Health Strategy (ESF) Centers, in the city of Itajubá, as well as in a philanthropic hospital and a teaching hospital in the city of Pouso Alegre, both located in the south of the State of Minas Gerais. These cities were chosen as they are centers of excellence in healthcare for the municipalities of the micro-region.

In the Primary Care Centers and Family Health Centers, the subjects were selected through a list containing the names of those who had chronic wounds, and their respective addresses. The same were enrolled in the hospital's outpatient center as they attended the unit.

As the inclusion criteria, the following were considered: individuals with chronic wounds of any etiology, for six weeks or over, with subjects being excluded if they showed an inability to understand or communicate verbally effectively. 

The sample size was calculated for a pilot-study, made up of 30 subjects, considering the tests of reliability and validity. A total was obtained of 200 individuals for the validation of the instrument, and 71 for the test and retest. 

Data collection took place in October 2012 - January 2013. The patients were invited to participate in the study and, after signing the Terms of Free and Informed Consent (TFIC), all the questionnaires were applied. 

After seven days, the FLQA-Wk, adapted to the Brazilian culture, was reapplied in 71 individuals with chronic wounds. The subjects of the retest were selected according to the order of arrival for applying the dressing in the outpatient center. 

The instruments used in data collection are indicated below.

- Sociodemographic and clinical questionnaire: this was developed specifically for the study, purely to characterize the individuals.
- Ferrans and Powers Quality of Life Index - Wound Version (FPQLI-WV): this contains 35 items, grouped into four interrelated domains. It addresses the fundamental aspects of the individuals with wounds: health and functioning, socioeconomic; psychological/spiritual and family^(5)^. The total score varies from zero (worst quality of life) to thirty (best quality of life). The instrument allows one to obtain five scores, that is, one for each domain and another for general quality of life.
*-* Adapted Freiburg Life Quality Assessment - Wound Module: the domains are calculated using the arithmetical mean of each response, following the re-codification of the "satisfaction" domain. The total score, on the other hand, is computed through the mean values of each domain^(9)^.

The questionnaire also presents three visual analog scales, graded from zero (very bad) to ten (very good). The individual measures her quality of life, health in general, and conditions of the wound in the previous week. This scale assists in controlling the values of the domains, that is, their values are compared with the instrument's total score. The higher the value of the score, the greater the negative influence in quality of life. The score varies from one (best quality of life) to five (worst quality of life). 

### Measuring of the psychometric properties 

The psychometric properties of the FLQA-Wk questionnaire were measured through reliability and validity. The questionnaire's reliability was assessed through two methods: internal consistency and stability. For the internal consistency, the Cronbach alpha was ascertained, and the stability was evaluated through the test-retest, with the Intraclass Correlation Coefficient (ICC) being calculated.

The validity was measured through the correlation of the values of the FLQA-Wk instrument's domains with those of the domains of the Ferrans and Powers Quality of Life Index - Wound Version (FPQLI-WV). In order to undertake correlations, the similarities of the contents between the domains were taken into consideration.

The correlation of the FLQA-Wk questionnaire's total score with the quality of life Visual Analog Scale (VAS) was also undertaken. 

The data were entered into the Microsoft Office 2007 Excel program and subsequently analyzed using the Faculty's Statistics Service, using the SAS statistical software, version 9.2. 

For the sociodemographic and clinical information, descriptive analysis was used for the continuous variables, and relative and absolute frequencies for the categorical variables. 

In order to analyze the Cronbach alpha, values of ≥0.70 were considered satisfactory^(^ 17 ^)^, and CCI values of >0.7 were considered acceptable to verify the instrument's stability^(^ 18 ^)^.

In the analysis of the validity, the Spearman correlation coefficient was used. For this, the following classification was adopted: close to 0.30, considered satisfactory; between 0.30 and 0.5, moderate magnitude; and above 0.5, strong magnitude^(^ 19 ^)^. The level of significance adopted for the statistical tests was 5%, that is, p value <0.05.

The present study was approved by the Research Ethics Committee of the Unicamp Faculty of Medical Sciences, under Opinion N. 44175.

## Results

### Cultural adaptation 

Cultural adaptation is a complex process, and with different methods recommended by the literature. In this study, this process followed the following phases: translation, synthesis of the translations, back translation, committee of specialists, pre-test and focus group^(^ 12 ^)^.

During the procedures of translation, synthesis of translation and back translation, complications did not occur. In the evaluation by the committee of specialists, taking into account that the rate of agreement between these was to be >0.80, the results indicated that the items were appropriate. 

Nevertheless, the committee suggested small changes in some questions, such as substitutions for synonyms, the inversion of phrases and the correction of spelling mistakes. In order to facilitate the reading and understanding, some of these suggestions were accepted.

During the undertaking of the pretest, some patients reported problems in understanding certain words, which impaired their understanding of questions. The term "insomnia" was cited by only one of the interviewees as difficult to understand and was not altered. The phrase "my leisure activities are restricted due to my condition" was selected by 33.3% due to the difficulty with the word "restricted", which was substituted with the term "reduced". 

The question of the social domain "limited the activities with other people" was not understood by 53.3% of the sample, because of the word "limited", which was changed to "reduced". The expression "physical activity is difficult for me" was interpreted erroneously by 66.6% of the participants, who stated it to be a synonym for physical exercise. However, the meaning of the phrase is related to physical effort. 

Through holding the focus group, the changes were discussed, with the Final Version of the Questionnaire (FVQ) being developed. This version was sent to one of the questionnaire's authors so that she could ascertain whether the content was compatible with the original. Her opinion was positive. 

### Characteristics of the study's subjects 

A total of 217 individuals with chronic wounds was recruited, of whom 17 did not meet the eligibility criteria. As a result, 200 individuals participated in the study (Table 1). 

**Table 1:** - Sociodemographic and clinical data. Itajubá and Pouso Alegre, State of Minas Gerais (MG), Brazil, 2013

	**N**	**%**	**Mean**	**Standard deviation**	
Age (years)			59.0	14.0	
Sex					
	Male	76	38.0		
	Female	124	62.0		
Marital situation					
	Married	85	42.5		
	Single	37	18.5		
	Widowed	53	26.5		
	Divorced	25	12.5		
Educational level					
	None	52	26.0		
	Basic education	97	48.5		
	High school	43	21.5		
	Higher education	8	4.0		
Number of wounds			1.5	0.9	
Duration of wounds (months)			34.7	65.0	
Type of wounds					
	Venous ulcer	90	45.0		
	Ischemic ulcer	20	10.0		
	Pressure ulcer	14	7.0		
	Diabetic ulcer	33	16.5		
	Mixed	43	21.5		

### Psychometric properties 

The adapted instrument (FLQA-Wk) presented a total Cronbach alpha of 0.86 (Table 2). 

**Table 2:** - Cronbach alpha of the domains of the FLQA-Wk* and of the total scale. Itajubá and Pouso Alegre, MG, Brazil, 2013

**Domains**	**Cronbach alpha**
Physical symptoms	0.63
Daily life	0.83
Social life	0.53
Psychological well-being	0.70
Treatment	0.68
Satisfaction	0.70
Total	0.86

*Freiburg Life Quality Assessment-Wound

In relation to stability, an Intraclass Correlation Coefficient (ICC) value of 0.93 was obtained (Confidence Interval: from 0.88 to 0.95).

The convergent validity appears with negative or inverse correlations, as the score of the FLQA-Wk indicates that the higher the score, the worse the quality of life, while in the score of the FPQLI-WV, the scoring is inverse: the higher the score, the better the quality of life. 

The correlations were of moderate and significant magnitudes between the values of the adapted instrument with those of the domains of the FPQLI-WV and with the Visual Analog Scale (Table 3).

**Table 3:** - Correlation of the FLQA-Wk with the FPQLI-WV and with the VAS. Itajubá and Pouso Alegre, MG, Brazil, 2013

**Domains** FLQA-Wk*	**VAS** **^†^** **Score**	**Domains of the FPQLI-WV** **^‡^**				
		**Health and functioning** **(p-value)**	**Psychological spiritual** **(p-value)**	**Family** **(p-value)**	**Socioeconomic** **(p-value)**	**Total score** **(p-value)**
Physical symptoms		-0.2696 (0.0001)				
Treatment		-0.3220 (<0.0001)				
Daily life		-0.3152 (<0.0001)				
Psychological well-being			-0.4415 (<0.0001)	-0.3778 (<0.0001)		-0.4008 (<0.0001)
Satisfaction			-0.4805 (<0.0001)		-0.4590 (<0.0001)	-0.3709 (<0.0001)
Social life		-0.2437 (0.0005)				
Total score	-0.3807 (<0.0001)	-0.4165 (<0.0001)				-0.3599 (<0.0001)

*Freiburg Life Quality Assessment-Wound †Visual Analog Scale ‡Ferrans and Powers Quality of Life Index - Wound Version

## Discussion

The subjects' characteristics were similar to those found in Brazilian and international publications, the same being observed in the study on the development of, and in the validation of, the Brazilian FPQLI-WV questionnaire^(^ 5 ^,^ 8 ^)^.

The sociodemographic data showed that 55.2% of the interviewees were female, with a mean age of 59.2 years; 47.7% were married, and the educational level of 60.4% was below basic education. Regarding the clinical characteristics of the wound, it was ascertained that 50.1% were of venous etiology, with a mean of 1.5 wounds per patient and mean duration of 51.1 months ^(^ 5 ^)^.

The data suggest that elderly women have a higher probability of developing chronic wounds, and that a low educational level is present in this population. This last characteristic seems to have hindered the application of the questionnaire, which underwent certain modifications in order to facilitate its understanding. 

In relation to the psychometric properties, the results show that the adapted version presented reliable and valid measurements for the population with chronic wounds, in the Brazilian culture. 

The homogeneity of the adapted version presented an acceptable Cronbach alpha, as proposed by Terwee, Bot and Boer, who consider values equal or superior to 0.7 to be adequate if the questionnaire is to be considered reliable^(^ 18 ^)^. High Cronbach alpha values, in instruments with multiple dimensions, indicate that the items are strongly related^(^ 20 ^)^.

A Cronbach alpha of 0.86 was also found in two studies with the FLQA-Wk questionnaire, involving patients with chronic and acute wounds. In one of the studies, the sample was one of 210 individuals treated with vacuum therapy, while the other encompassed 510 patients with chronic wounds of different etiologies, in the lower limbs treated with different therapies^(^ 9 ^)^.

In one study undertaken in Germany, with 198 individuals with wounds of a venous origin, an internal consistency for the FLQA-Wk of 0.87 was obtained^(^ 9 ^)^.

The stability of the questionnaire adapted to Portuguese presented an intraclass correlation coefficient value of 0.93, which can be considered excellent^(^ 18 ^)^.

In one randomized study, which consisted of the measurement of the quality of life of patients with venous ulcers, receiving treatment with keratinocyte transplants and with local compression, a test-retest value of 0.69 was observed, which can be considered to be of moderate magnitude^(^ 9 ^)^.

It is worth emphasizing that, in the present study, the interval of the application of the instrument was of seven days, as it was considered that, after this period, changes could occur in the conditions of the wound, interfering in the quality of life. In the original validation study, a period of four weeks was used. The adapted questionnaire showed temporal stability when applied at separate times for the same population in similar conditions. 

In relation to validity, the values of the correlations of the FLQA-Wk with those of the FPQLI-WV were shown to be significant. The FPQLI-WV questionnaire is one of the few in Brazil which was developed and validated specifically for assessing the quality of life of patients with chronic and acute wounds^(^ 5 ^)^.

The FPQLI-WV was shown to be an instrument capable of measuring the quality of life of patients with chronic wounds and diabetic foot, making it possible for the nurse to provide quality interventions, with a view to assessing not only the wound, but also the individual as a whole^(^ 5 ^,^ 21 ^)^.

The study on the construction and validation of the FPQLI-WV presented reliability and validity which were adequate for assessing general quality of life, the health, and the psychological and spiritual aspects of individuals with acute or chronic wounds^(^ 5 ^)^, conferring on the FLQA-Wk the capacity to measure the quality of life of patients with chronic wounds. 

In relation to the correlation of the FLQA-Wk total score with the quality of life score of the visual analog scale, the value obtained was considered to be of moderate magnitude (-0.38) and significant. 

The same correlation was undertaken in three separate studies with the original instrument, with values being found of: -0.38 (p=0.001), -0.67 (p=0.001) and -0.61 (p=0.001), considered to be of moderate and strong magnitude^(^ 9 ^)^.

In this way, it is hoped that the cultural adaptation of the Freiburg Life Quality Assessment-Wound module allows the attendance to these patients to be undertaken in a holistic way, also taking into account their quality of life, and contributing to quality care^(^ 22 ^)^.

It is emphasized that the original version was developed to be self-applied or to be answered through interview. The FLQA-Wk questionnaire presented satisfactory psychometric properties in order for it to be applied in individuals with chronic wounds of different etiologies, and used both in the clinical area and in research. 

## Conclusions

The cultural adaptation of the Freiburg Life Quality Assessment-Wound module was undertaken following the international methodology, resulting in a reliable version. In the data collection procedure, the instrument was revealed to be easy to understand and apply. 

The questionnaire also presented satisfactory internal consistency and temporal stability. In relation to its validity, the adapted FLQA-Wk questionnaire demonstrated correlations of moderate and significant magnitudes with the values of the Ferrans and Powers Quality of Life Index - Wound Version questionnaire.

The present scale has the potential to take on a predominant role in the study of the quality of life of persons with chronic wounds in Brazil, and must be used in research contexts, as well as in clinical situations and as an instrument to assist in diagnosis and care, involving the professionals from the health area in a multi-professional way.
